# Evaluation of ranibizumab and aflibercept for the treatment of diabetic macular edema in daily clinical practice

**DOI:** 10.1371/journal.pone.0223793

**Published:** 2019-10-23

**Authors:** Pablo Plaza-Ramos, Enrique Borque, Alfredo García-Layana

**Affiliations:** 1 Ophthalmology Department, Complejo Hospitalario de Navarra, Pamplona, Spain; 2 Ophthalmology Department, Clínica Universitaria de Navarra, Pamplona, Spain; Massachusetts Eye & Ear Infirmary, Harvard Medical School, UNITED STATES

## Abstract

**Purpose:**

To evaluate the efficacy and safety of ranibizumab and aflibercept in the treatment of diabetic macular edema in a real world study, and to compare the two treatments with each other.

**Methods:**

Retrospective observational study of 213 eyes from 141 patients with diabetic macular edema was completed between June 2014 and June 2016. 122 were treated with ranibizumab intravitreal injection and 91 with aflibercept intravitreal injection, with a loading phase of 3 injections and a Pro Re Nata protocol. The drug was selected by the physician and fluorescein angiography was performed by physician`s criteria. Re-treatment was performed when a decline in BCVA, an increase of central macular thickness or an increase or persistence of intraretinal fluid in OCT was observed. The primary outcome was the mean change in best corrected visual acuity at 1 year, while central macular thickness, central macular volume, the number of injections and visits were evaluated as secondary outcomes. The correlation between BCVA at 4^th^ month visit and BCVA at 12^th^ month visit was also evaluated.

**Results:**

The mean baseline best corrected visual acuity for the eyes treated with ranibizumab was 0.55 (+/- 0.35) logMAR, and with aflibercept it was 0.48 (+/- 0.29) (P = 0.109). Best corrected visual acuity improved in both groups, and at the end of the follow-up was 0.40 (+/- 0.35) in the ranibizumab group and 0.40 (+/- 0.29) in the aflibercept group (P = 0.864). Best corrected visual acuity at 4^th^ month visit is correlated at a high value (R = 0.789) with the one at the end of the study. No differences were found in central macular thickness, central macular volume and glycosylated hemoglobin when adjusting with baseline values. The overall number of injections was 5.77 (+/- 2.01), being 5.56 (+/- 2.0) in the ranibizumab group and 6.07 (+/- 1.99) in the aflibercept group (P = 0.069). The main outcome determining final best corrected visual acuity was the baseline best corrected visual acuity (P<0.001).

**Conclusion:**

There are no differences in efficacy between ranibizumab and aflibercept in diabetic macular edema treatment in this real world study.

## Introduction

Diabetic macular edema (DME) is one of the leading causes of visual impairment in the working-age population in developed countries [1]. It is characterized by exudation and accumulation of extracellular fluid in the macula, secondary to an increase in vascular permeability [2], and hyperglycemia is the main factor in its development [3].

In the past, treatment options for DME were limited to macular laser photocoagulation, intravitreal triamcinolone, and pars plana vitrectomy, in addition to adequate systemic control of diabetes and hypertension [1].

Vascular endothelial growth factor (VEGF) is a protein that promotes angiogenesis and increases vascular permeability. VEGF is a main cause of vascular growth and edema, and is present in many vascular diseases such as DME [4]).

Intravitreal anti-VEGF medications are recognized as improving visual outcomes and decreasing macular fluid in patients with DME [5]. Ranibizumab (Lucentis; Genentech, South San Francisco, CA), was the first of these to be licensed by the European Medicines Agency (EMA) in 2011. It is a humanized monoclonal antibody Fab fragment specifically designed for ocular use. It binds to VEGF-A with high affinity and inhibits all isoforms of VEGF-A [1,6–9]. Aflibercept (Eylea; Regeneron, Tarrytown, NY) was licensed by the EMA in 2014. It is a soluble decoy receptor fusion protein that inhibits PIGF in addition to all isoforms of VEGF-A and VEGF-B [2,10–12].

Multiple phase 3 studies have confirmed that anti-VEGF treatment results in improved visual and anatomic outcomes after the first year of treatment [6,7,10,13]. Although these results may not be permanent, and multiple injections may be required to maintain treatment efficacy, the maintenance of one-year visual gains and a reduced frequency of injections in following years have been demonstrated [8,9,13,14].

The Diabetic Retinopathy Clinical Research Network (DRCRnet) Protocol T showed that treatment with intravitreal bevacizumab (Avastin; Genentech, South San Francisco, CA), ranibizumab, and aflibercept resulted in improvements in visual and anatomic outcomes over baseline [13,15]. This was the first trial comparing the three treatments with each other.

Results at 1 year showed that the mean change in best-corrected visual acuity (BCVA) primary outcome varied based on the baseline BCVA initially presenting. Intravitreal aflibercept injection demonstrated superiority in patients with 20/50 and worse baseline BCVA, whereas no clinically significant difference was seen across treatment groups when the initial BCVA was 20/40 or better [13].

However, results at Year 2 showed no difference between ranibizumab and aflibercept in patients with an initial BCVA of 20/50 or worse, with both being superior to bevacizumab. No differences where shown in patients with an initial BCVA of 20/40 or better [16].

Routine clinical practice does not always replicate the findings found in clinical research trials, since these studies enroll patients with strict inclusion and exclusion criteria that do not reflect the breadth of patients seen in routine clinical practice [15]. The intensive treatment schedules and close monitoring typically employed in clinical trials may result in selected populations that do not reflect routine clinical practice. These differences cause discrepancies between real life outcome and those reported in clinical trials [14].

Up to now, a limited amount of data is available on real-world clinical experiences in a large patient population treated with the two drugs available and approved by the Food and Drug Administration (FDA) and the European Medicines Agency (EMA), aflibercept and ranibizumab.

The purpose of this study is to evaluate the efficacy of aflibercept and ranibizumab, for the treatment of DME in routine clinical practice, and to compare them, as well as establishing patterns of good and bad response to both anti-VEGF treatments.

## Methods

### Study design

This was a retrospective, single-center, 12-month observational study carried-out in a tertiary referral hospital. The study was approved by both the ethics committee of Complejo Hospitalario de Navarra (CHN) and the University of Navarre, in Pamplona, Spain. It was conducted in accordance with the principles of the Helsinki Declaration, and written informed consent was obtained from all the individual participants included in the study.

### Study participants

Patients of both sexes, aged >18 years and clinically diagnosed of DME confirmed both, clinically and with OCT, were enrolled in the study. They were treated with ranibizumab or aflibercept for at least 1 year of follow-up, starting between June 2014 and June 2016.

The selection of the anti-VEGF drug was made by the physician according to his own criteria.

Patients treated with previous intravitreal injections of aflibercept, bevacizumab or ranibizumab within 3 months before the start of the study were excluded. Patients treated with intravitreal injections of triamcinolone or dexamethasone within 6 months before the start of the study were also excluded, as well as patients with vascular retinal diseases other than diabetic retinopathy, high myopia (>6D), chorioretinitis or any other fundus disease associated with morphologic and functional changes.

The primary endpoint was to evaluate the efficacy in terms of change in BCVA of both anti-VEGF agents used for DME and approved by the EMA in a real world study. Secondary endpoints were: to compare ranibizumab and aflibercept with each other functionally and anatomically, to evaluate the response to anti-VEGF therapy after the loading dose, and to correlate the results with those at the end of the study; to evaluate the response to anti-VEGF treatment according to systemic control; to evaluate the response to anti-VEGF treatment according to baseline BCVA and to analyze the features of patients with poor BCVA at the end of the study. We also divided the sample in naïve or non-naïve patients to compare both groups between them.

### Study procedures

As in routine clinical practice, at baseline, the patients diagnosed with DME underwent a complete ophthalmologic examination, including medical history; BCVA assessed with Snellen visual charts, slit lamp biomicroscopy, intraocular pressure (IOP) evaluation and dilated fundus examination. Spectral domain optical coherence tomography (SD-OCT; Heidelberg Engineering, Heidelberg, Germany) was also performed. The same OCT was used throughout the study period.

Every patient was treated with a loading dose of three intravitreal injections and was followed-up with a PRN regimen.

The follow-up examinations included BCVA evaluation, slit-lamp biomicroscopy, fundus examination and OCT. Re-treatment criteria followed a BCVA and OCT-driven regimen aimed at achieving complete macular fluid regression and BCVA stability. Re-treatment was performed when a decline in BCVA, an increase of central macular thickness or an increase or persistence of intraretinal fluid in OCT was observed. Furthermore, the date of the expected and the real visit was compiled, and systemic control was also evaluated.

Focal and grid laser macular treatment was registered, and was determined by the treating clinician, as well as PRP and cataract surgery.

BCVA was assessed using Snellen visual charts and then converted to a logarithm of the minimum angle of resolution (logMAR) units for statistical analysis, as is usually done in most clinical studies for analyzing retrospective data collections.

Central macular thickness was defined as the average thickness within the central 1000 μm diameter of the Early Treatment Diabetic retinopathy Study (ETDRS) grid, using the macular thickness map provided by the HRA software.

In order to divide groups with bad BCVA and good BCVA, in the bad BCVA group we included all the patients with a baseline BCVA of 0.4 logMAR or higher, and in the good BCVA group, all the rest, given that 0.4 logMAR was the median of baseline BCVA in our sample.

### Statistics

Data was recorded with the Microsoft Excel 2010 program and added to IBM SPPSS Statistics 25 for the analysis. Shapiro-Wilk and the Kolmogorov- Smirnov tests were employed to check normality.

Results are expressed as mean and standard deviation (SD) if the variables are continuous and with frequencies and percentages if the variables are categorical. Baseline variables were compared between treatment groups using student’s t test and Mann-Whitney U test in the continuous cases and with Chi-squared and Fisher test for categorical variables. Lineal univariate regression models were fitted with baseline visual acuity as the dependent variable. The variables with baseline differences between treatment groups, variables associated with the baseline visual acuity, those ones statistically different in the univariate analysis and relevant variables were included in a linear multivariate model. Furthermore, the linear correlation between continuous variables was assessed by the Pearson correlation coefficient (r). Statistical significance was set at P<0.05.

## Results

### Basal characteristics

213 eyes from 141 patients were enrolled in the study and were followed-up for 12 months. 122 were treated with ranibizumab and 91 with aflibercept. Baseline characteristics and demographic data for the cohort are summarized in Table 1. No differences were appreciated in terms of age, sex, laterality and type of DM. 104 patients were considered non-naïve, 37 patients in the ranibizumab-treated group (30.6% of the total patients of this group), and 67 patients in the aflibercept-treated group (70.6% of the total patients of this group) (P<0.001). Basal BCVA was 0.55 (+/- 0.35) in the ranibizumab-treated group and 0.48 (+/-0.29) in the aflibercept-treated group (P = 0.109). Central macular thickness (CMT) was 483.45 (+/-142.13) μm in the ranibizumab-treated group and 419.46 (+/-104.61) μm in the aflibercept-treated group (P<0.001). In addition, central macular volume (CMV) turned out to be 10.73 (+/-2.21) mm^3^ in the ranibizumab-treated group and 9.63 (+/-1.35) mm^3^ in the aflibercept-treated group (P<0.001).

**Table 1:** Patients basal characteristics.

	Study population (n = 213)	Ranibizumab (n = 122)	Aflibercept (n = 91)	P
**Demographics**				
**Age, mean ± SD, years**	**69.05 (+/- 10.58)**	**69.30 (+/- 10.34)**	**68.71 (+/- 10.94)**	**.690**
**Females, % (n)**	**31.5% (n = 67)**	**29.5% (n = 36)**	**34.1% (n = 31)**	**.313**
**Right eye, % (n)**	**50.7% (n = 108)**	**52.4% (n = 64)**	**48.5% (n = 44)**	**.649**
**Diabetes Mellitus**				
**Type I, % (n)**	**7.5% (n = 16)**	**7.4% (n = 9)**	**7.7% (n = 7)**	**1**
**Type II, % (n)**	**90.1% (192)**	**90.2% (n = 110)**	**90.2% (n = 82)**	**1**
**LADA, % (n)**	**2.4% (n = 5)**	**2.4% (n = 3)**	**2.1% (n = 2)**	**1**
**Duration, mean ± SD, years**	**16.73 (+/- 8.96)**	**15.89 (+/- 9.29)**	**17.86 (+/- 8.41)**	**.110**
**Metabolic factors**				
**HbA1c, mean ± SD, %**	**7.88 (+/- 1.63)**	**7.91 (+/- 1.27)**	**7.84 (+/- 2.03)**	**.230**
**Colesterol, mean ± SD, mg/dl**	**181.17 (+/- 44.44)**	**186.76 (+/- 43.91)**	**176.67 (+/- 36.40)**	**.070**
**Creatinine, mean ± SD, mg/dl**	**91.51 (+/- 65.40)**	**88.56 (+/- 53.27)**	**95.46 (+/- 78.94)**	**.870**
**Microalbuminuria, mean ± SD, mg/day**	**232.02 (+/- 567.30)**	**236.84 (+/- 586.58)**	**225.57 (+/- 543.52)**	**.040**
**Visits, mean ± SD**	**8.43 (+/- 1.56)**	**8.55 (+/- 1.54)**	**8.27 (+/- 1.59)**	**.205**
**Inyections, mean ± SD**	**5.77 (+/- 2.01)**	**5.56 (+/- 2.0)**	**6.07 (+/- 1.99)**	**.069**
**Mean baseline BCVA, mean ± SD, logMar**	**0.52 (+/- 0.34)**	**0.55 (+/- 0.35)**	**0.48 (+/- 0.29)**	**.109**
**Mean baseline CMT, mean ± SD, μm**	**456.11 (+/- 131.08)**	**483.45 (+/- 142.13)**	**419.46 (+/- 104.61)**	**.000**

(PRP: panretinalphotocoagulation, PPV: pars plana vitrectomy, HbA1c: glycosylated haemoglobin, BCVA: best corrected visual acuity, CMT: central macular thickness, CMV: central macular volume)

The overall mean for intravitreal injections was 5.77 (+/- 2.01). The ranibizumab-treated group received 5.56 (+/- 2.0) injections, whereas the aflibercept-treated group received 6.07 (+/- 1.99) injections (P = 0.069).

### Global results

The overall results of the population are resumed in Table 2. We can appreciate that baseline HbA1c was 7.88 (+/-1.63) %, and it decreased to 7.80 (+/-1.50) % at 4^th^ month visit, and to 7.79 (+/-1.43) at the end of the study. With regard to BCVA, we can appreciate baseline overall BCVA was 0.52 (+/-0.34) logMAR, BCVA at 4^th^ month visit was 0.40 (+/-0.31) logMAR, and BCVA at the end of the study was 0.40 (+/-0.33) logMAR. According to CMT, the baseline value was 456.11 (+/- 131.08) μm, CMT at 4^th^ month visit was 374.04 (+/-99.99) μm and CMT at the end of the study was 372.15 (+/-111.76). Finally, in regards to CMV, the baseline value was 10.26 mm^2^ (+/-1.96) mm^2^, CMV at 4^th^ month visit was 9.40 (+/-1.29) mm^2^ and CMV at the end of the study was 9.49 (+/-1.24) mm^2^.

**Table 2:** Overall results.

	Study population (n = 213)
**HbA1c**	
**Baseline, mean ± SD, %**	**7.88 (+/- 1.63)**
**4th Month, mean ± SD, %**	**7.80 (+/- 1.50)**
**12th Month, mean ± SD, %**	**7.79 (+/- 1.43)**
**BCVA**	
**Baseline, mean ± SD, logMar**	**0.52 (+/- 0.34)**
**4th Month, mean ± SD, logMar**	**0.40 (+/- 0.31)**
**12th Month, mean ± SD, logMar**	**0.40 (+/- 0.33)**
**CMT**	
**Baseline, mean ± SD, μm**	**456.11 (+/-131.08)**
**4th Month, mean ± SD, μm**	**374.04 (+/-99.99)**
**12th Month, mean ± SD, μm**	**372.15 (+/-111.76)**
**CMV**	
**Baseline, mean ± SD, mm^3^**	**10.26 (+/- 1.96)**
**4th Month, mean ± SD, mm^3^**	**9.40 (+/- 1.29)**
**12th Month, mean ± SD, mm^3^**	**9.49 (+/- 1.24)**

(HbA1c: glycosylated haemoglobin, BCVA: best corrected visual acuity, CMT: central macular thickness, CMV: central macular volume)

We performed, then, a lineal univariate regression with the final BCVA as dependent value and which is resumed in Table 3. Many variables were statistically significant, like age (P<0.001), sex (P<0.001), previous treatment with bevacizumab (P<0.001), previous treatment with ranibizumab (P = 0.002), previous treatment with dexamethasone (P = 0.031), previous PRP (P<0.001), previous cataract surgery (P<0.001) and previous pars plana vitrectomy (P = 0.011), but the most important one was baseline BCVA (P<0.001).

**Table 3:** Lineal univariate regression.

	Category	Beta (IC 95%)	P
**Drug**	**Ranibizumab**	**Reference**	
	**Aflibercept**	**0.008 (-0.082, 0.097)**	**.864**
**Age**		**0.083 (0.004, 0.012)**	**.000**
**Sex**	**Male**	**Reference**	
	**Female**	**0.216 (0.125, 0.307)**	**.000**
**Previous Bevacizumab**	**No**	**Reference**	
	**Yes**	**0.205 (0.092, 0.317)**	**.000**
**Previous Ranibizumab**	**No**	**Reference**	
	**Yes**	**0.144 (0.054, 0.234)**	**.002**
**Previous Dexamethasone**	**No**	**Reference**	
	**Yes**	**0.178 (0.016, 0.340)**	**.031**
**Previous focal laser**	**No**	**Reference**	
	**Yes**	**-0.061 (-0.185, 0.063)**	**.334**
**Previous PRP**	**No**	**Reference**	
	**Yes**	**0.206 (0.121, 0.290)**	**.000**
**Previous macular laser**	**No**	**Reference**	
	**Yes**	**0.329 (0.220, 0.439)**	**.000**
**Previous Cataract surgery**	**No**	**Reference**	
	**Yes**	**0.204 (0.104, 0.305)**	**.000**
**Previous VPP**	**No**	**Reference**	
	**Yes**	**0.199 (0.046, 0.352)**	**.011**
**Baseline BCVA**	**No**	**Reference**	
	**Yes**	**0.679 (0.585, 0.779)**	**.000**
**Baseline CMT**	**No**	**Reference**	
	**Yes**	**0.026 (-0.007, 0.060)**	**.126**
**Baseline CMV**	**No**	**Reference**	
	**Yes**	**0.021 (-0.002, 0.042)**	**.070**

(PRP: panretinalphotocoagulation, PPV: pars plana vitrectomy, BCVA: best corrected visual acuity, CMT: central macular thickness, CMV: central macular volume)

The predictive value of final BCVA was analyzed afterwards with lineal multivariate regression with the parameters shown in Table 4. The model is adjusted by the baseline BCVA, which was shown as the most correlated with the final BCVA. In spite of that, several influential factors were found anyway and can be appreciated in Table 4. We appreciate that female sex (P = 0.010), the fact of being previously treated with bevacizumab (P = 0.001) and with macular laser (P = 0.041) were three influential factors showed as statistically significant. The model shows no differences between patients treated with one or the other drug that concern us (P = 0.587).

**Table 4:** Multivariate model of final BCVA adjusted by basal BCVA.

	Category	Beta (IC 95%)	P
**Drug**	**Ranibizumab**	**Reference**	
	**Aflibercept**	**-0.021 (-0.097, 0.055)**	**.587**
**Age**		**0,003 (-0.001, 0.006)**	**.125**
**Sex**	**Male**	**Reference**	
	**Female**	**0.088 (0.021, 0.154)**	**.010**
**Previous Bevacizumab**	**No**	**Reference**	
	**Yes**	**0.145 (0.057, 0.233)**	**.001**
**Previous Ranibizumab**	**No**	**Reference**	
	**Yes**	**0.048 (-0.039, 0.135)**	**.282**
**Previous Dexamethasone**	**No**	**Reference**	
	**Yes**	**-0.044 (-0.173, 0.086)**	**.508**
**Previous focal laser**	**No**	**Reference**	
	**Yes**	**-0.030 (-0.115, 0.055)**	**.484**
**Previous PRP**	**No**	**Reference**	
	**Yes**	**0.042 (-0.024, 0.107)**	**.212**
**Previous macular laser**	**No**	**Reference**	
	**Yes**	**0.095 (0.004, 0.187)**	**.041**
**Previous Cataract surgery**	**No**	**Reference**	
	**Yes**	**0.005 (-0.080, 0.090)**	**.905**
**Previous VPP**	**No**	**Reference**	
	**Yes**	**0.015 (-0.097, 0.127)**	**.792**

(PRP: panretinalphotocoagulation, PPV: pars plana vitrectomy)

We performed also a comparison between naïve and non-naïve patients, where we can appreciate that naïve patients received 5.83 (+/- 2.02) injections and non-naïve patients 5.71 (+/- 2.01) injections (P = 0.656). Moreover, naïve patients started with a baseline BCVA of 0.45 (+/-0.28) logMAR, and the non-naïve patients with a BCVA of 0.58 (+/-0.38) logMAR (P<0.001). The naïve group of patients ended up with a BCVA of 0.27 (+/-0.18) logMAR and the non-naïve group ended up with a BCVA of 0.54 (+/-0.27) logMAR (P<0.001). When comparing CMT, naïve patients started up with 480.73 (+/-142.72) μm and non-naïve patients with 430.31 (+/-112.67) μm (P<0.001). At the end of the study naïve patients ended up with a CMT of 362.56 (+/-96.01) μm, and non-naïve patients with a CMT of 382.19 (+/-125.88) μm (P = 0.800). These results are summarized in Table 5.

**Table 5:** Change from baseline to 4^th^ month and to 12^th^ month in naïve and non-naïve patients.

	Study population (n = 213)	Naïve (n = 109)	Non-Naïve (n = 104)	P
**Inyections, mean ± SD**	**5.77 (+/- 2.01)**	**5.56 (+/- 2.0)**	**6.07 (+/- 1.99)**	**.069**
**BCVA**				
**Baseline, mean ± SD, logMar**	**0.52 (+/- 0.34)**	**0.45 (+/- 0.28)**	**0.58 (+/- 0.38)**	**.000**
**4th Month, mean ± SD, logMar**	**0.40 (+/- 0.31)**	**0.30 (+/- 0.21)**	**0.51 (+/- 0.37)**	**.000**
**12th Month, mean ± SD, logMar**	**0.40 (+/- 0.33)**	**0.27 (+/- 0.18)**	**0.54 (+/- 0.38)**	**.000**
**CMT**				
**Baseline, mean ± SD, μm**	**456.11 (+/-131.08)**	**480.73 (+/-142.72)**	**430.31 (+/- 112.67)**	**.000**
**4th Month, mean ± SD, μm**	**374.04 (+/-99.99)**	**370.33 (+/-94.33)**	**378.19 (+/- 105.91)**	**.005**
**12th Month, mean ± SD, μm**	**372.15 (+/-111.76)**	**362.56 (+/-96.01)**	**382.19 (+/- 125.88)**	**.800**

(BCVA: best corrected visual acuity, CMT: central macular thickness)

### Ranibizumab vs aflibercept

With regard to the comparison between the ranibizumab-treated group and the aflibercept-treated group, we can see that BCVA at 4^th^ month visit was 0.41 (+/- 0.34) logMAR in patients treated with ranibizumab and 0.40 (+/- 0.27) logMAR in those treated with aflibercept (P = 0.888). At the end of the study, BCVA remained at 0.40 (+/- 0.35) logMAR in the ranibizumab group, and at 0.40 (+/- 0.29) log-Mar (P = 0.864) in the patients treated with aflibercept. CMT was 388.67 (+/-100.71) μm at 4^th^ month visit in the ranibizumab-treated group, and 354.73 (+/-96.16) μm in the aflibercept-treated group at that visit (P = 0.014). At the end of the study, the ranibizumab-treated group treatment group achieved 377.06 (+/-116.59) μm of CMT, and the aflibercept-tretaed group maintained 365.56 (+/-105.22) μm (P = 0.459). Regarding the change of CMT we can appreciate that patients in the ranibizumab-treated group reduced their CMT 106.39 (+/-166.86) μm, whereas patients in the aflibercept-treated group experimented a reduction of 53.90 (+/-119.15) μm (P = 0.011).The of decrease CMV progression was comparable to that of CMT, as can be seen in Table 6. Lastly, with regard to HbA1c, no differences were perceived either at basal visit or at 4^th^ month visit and at 12^th^ month visit. These results are summarized in Table 6.

**Table 6:** Change from baseline to 4^th^ month and to 12^th^ month.

	Study population (n = 213)	Ranibizumab (n = 122)	Aflibercept (n = 91)	P
**HbA1c**				
**Baseline, mean ± SD, %**	**7.88 (+/- 1.63)**	**7.91 (+/- 1.27)**	**7.84 (+/- 2.03)**	**.230**
**4**^**th**^ **Month, mean ± SD, %**	**7.80 (+/- 1.50)**	**7.86 (+/- 1.34)**	**7.72 (+/- 1.70)**	**.319**
**12**^**th**^ **Month, mean ± SD, %**	**7.79 (+/- 1.43)**	**7.94 (+/- 1.20)**	**7.58 (+/- 1.68)**	**.060**
**BCVA**				
**Baseline, mean ± SD, logMar**	**0.52 (+/- 0.34)**	**0.55 (+/- 0.35)**	**0.48 (+/- 0.29)**	**.109**
**4**^**th**^ **Month, mean ± SD, logMar**	**0.40 (+/- 0.31)**	**0.41 (+/- 0.34)**	**0.40 (+/- 0.27)**	**.888**
**12**^**th**^ **Month, mean ± SD, logMar**	**0.40 (+/- 0.33)**	**0.40 (+/- 0.35)**	**0.40 (+/- 0.29)**	**.864**
**CMT**				
**Baseline, mean ± SD, μm**	**456.11 (+/-131.08)**	**483.45 (+/- 142.13)**	**419.46 (+/- 104.61)**	**.000**
**4**^**th**^ **Month, mean ± SD, μm**	**374.04 (+/-99.99)**	**388.67 (+/- 100.71)**	**354.73 (+/- 96.16)**	**.014**
**12**^**th**^ **Month, mean ± SD, μm**	**372.15 (+/-111.76)**	**377.06 (+/- 116.59)**	**365.56 (+/- 105.22)**	**.459**
**Change, mean ± SD, μm**	**-83.97 (+/- 150.32)**	**-106.39 (+/- 166.86**	**-53.90 (+/- 119.15)**	**.011**
**CMV**				
**Baseline, mean ± SD, mm^3^**	**10.26 (+/- 1.96)**	**10.73 (+/- 2.21)**	**9.63 (+/- 1.35)**	**.000**
**4**^**th**^ **Month, mean ± SD, mm^3^**	**9.40 (+/- 1.29)**	**9.67 (+/- 1.49)**	**9.05 (+/- 0.85)**	**.001**
**12**^**th**^ **Month, mean ± SD, mm^3^**	**9.49 (+/- 1.24)**	**9.69 (+/- 1.61)**	**9.21 (+/- 1.28)**	**.024**
**Change, mean ± SD, mm^3^**	**-0.77 (+/- 1.84)**	**-1.04 (+/- 2.02)**	**-0.42 (+/- 1.51)**	**.015**

(HbA1c: glycosylated haemoglobin, BCVA: best corrected visual acuity, CMT: central macular thickness, CMV: central macular volume)

We checked, also, the correlation between BCVA at the end of the study with the one showed at the 4^th^ month visit, which turned out to be very high (R = 0.789; P<0.000).

### Good baseline BCVA vs bad baseline BCVA

Moreover, we performed a comparison between patients in both groups according to their basal BCVA in logMAR. Patients with 0.4 logMAR or higher values were clustered in the group known as the bad BCVA group. On the contrary, patients with lower basal values of 0.4 logMAR BCVA were considered to be part of the good BCVA group. Patients in the group with good BCVA at baseline started out with 0.24 (+/- 0.78) logMAR, whereas the bad BCVA group started out with 0.69 (+/- 0.32) logMAR (P<0.001). At 4^th^ month visit patients from the good baseline BCVA group had 0.21 (+/- 0.13) logMAR and the patients from the bad baseline BCVA had 0.52 (+/- 0.30) logMAR (P<0.001). At the end of the study the patients with good baseline BCVA had 0.22 (+/- 0.19) logMAR, and the patients with bad baseline BCVA had 0.51 (+/- 0.35) logMAR (P<0.001). In terms of CMT and CMV, differences are present at the beginning of the study, but they disappear at 4^th^ month and 12^th^ month visits, as can be observed in Table 7. In terms of HbA1c, values were higher in the group with good basal BCVA, and those differences are statistically significant in the baseline visit and the 4^th^ month visit, disappearing in the final visit. Those results are summarized in Table 7.

**Table 7:** Change from baseline to 4^th^ month and to 12^th^ month in patients with good and bad basal BCVA.

	Study population (n = 213)	Good BCVA (n = 80)	Bad BCVA (n = 133)	P
**HbA1c**				
**Baseline, mean ± SD, %**	**7.88 (+/- 1.63)**	**8.22 (+/- 1.77)**	**7.68 (+/- 1.55)**	**.006**
**4th Month, mean ± SD, %**	**7.80 (+/- 1.50)**	**8.12 (+/- 1.64)**	**7.61 (+/- 1.37)**	**.015**
**12th Month, mean ± SD, %**	**7.79 (+/- 1.43)**	**8.01 (+/- 1.56)**	**7.65 (+/- 1.33)**	**.092**
**BCVA**				
**Baseline, mean ± SD, logMar**	**0.52 (+/- 0.34)**	**0.24 (+/- 0.78)**	**0.69 (+/- 0.32)**	**.000**
**4th Month, mean ± SD, logMar**	**0.40 (+/- 0.31)**	**0.21 (+/- 0.13)**	**0.52 (+/- 0.30)**	**.000**
**12th Month, mean ± SD, logMar**	**0.40 (+/- 0.33)**	**0.22 (+/- 0.19)**	**0.51 (+/- 0.35)**	**.000**
**CMT**				
**Baseline, mean ± SD, μm**	**456.11 (+/-131.08)**	**412.31 (+/-89.02)**	**482.46 (+/- 144.88)**	**.000**
**4th Month, mean ± SD, μm**	**374.04 (+/-99.99)**	**362.48 (+/-88.63)**	**381.20 (+/- 105.94)**	**.186**
**12th Month, mean ± SD, μm**	**372.15 (+/-111.76)**	**366.34 (+/-88.90)**	**375.64 (+/- 123.70)**	**.558**
**CMV**				
**Baseline, mean ± SD, mm^3^**	**10.26 (+/- 1.96)**	**9.72 (+/- 1.05)**	**10.58 (+/- 2.29)**	**.022**
**4th Month, mean ± SD, mm^3^**	**9.40 (+/- 1.29)**	**9.22 (+/- 0.84)**	**9.51 (+/- 1.49)**	**.429**
**12th Month, mean ± SD, mm^3^**	**9.49 (+/- 1.24)**	**9.46 (+/- 1.61)**	**9.50 (+/- 1.63)**	**.985**

(HbA1c: glycosylated haemoglobin, BCVA: best corrected visual acuity, CMT: central macular thickness, CMV: central macular volume)

We registered other treatments during the study, such as focal and grid laser, PRP and cataract surgery. 0 patients of both groups were treated with grid laser (P = 1.000). 11 patients were treated with focal laser in the ranibizumab-treated group and 3 patients in the aflibercept group (P = 0.096). 8 patients were treated with PRP in the ranibizumab group and 5 in the aflibercept group (P = 0.975). Lastly, 7 patients were operated for cataracts in the ranibizumab group, and 2 of them in the aflibercept group (P = 0.306). No differences were observed in any comparison.

No serious complications such as retinal detachment or endophthalmitis were detected in any patient in the study during the follow-up period.

## Discussion

DME is caused by excessive retinal vascular permeability, leading to the accumulation of fluid in the retina and increasing its thickness. This event is associated with the breakdown of the blood retinal barrier and the increase in the levels of VEGF [2,4]. The development of VEGF inhibitors (anti-VEGF) has revolutionized DME treatment, with an improvement in visual acuity and a reduction of CMT, as several randomized clinical trials attest [6,7,10,13]. However, there are only a few studies concerning this issue in real world studies with more than 200 eyes included, and the majority of them have included a very low number of patients [1,2,4,14,15,17].

Protocol T was the first randomized clinical trial that compared ranibizumab and aflibercept with each other. First year aflibercept seems to be more efficient in terms of improving visual acuity and reducing CMT, especially in eyes with low BCVA [13], but in the second year of analysis these differences disappeared [16]. Subsequently, the question was whether the differences observed in the first year of the study remain in real world studies.

On the one hand, statistics proved that baseline BCVA is not different between ranibizumab and aflibercept groups (P = 0.109). The ranibizumab-treated group had a baseline BCVA of 0.55 (+/-0.35) logMAR and the aflibercept-treated one had a baseline BCVA of 0.48 (+/- 0.29) logMAR. An important issue to take into account is the difference between the numbers of naïve patients in both groups. In the ranibizumab-treated group, 85 eyes were naïve (69.7%), whereas in the aflibercept-treated group only 24 patients were naïve (26.3%). It has already been proven that patients with chronic DME who have previously been treated have a poorer prognosis and a limited visual improvement [6,9,10,14,18], and we can appreciate this issue also in our study and can explain that the change in BCVA between both drugs even though is not statistically different it may seem that is greater in the ranbizumab group. Despite this, there is no difference between BCVA comparing ranibizumab and aflibecept at the end of the follow-up, with it being 0.40 (+/- 0.35) logMAR in the ranibizumab-treated group, and 0.40 (+/- 0.29) logMAR in the aflibercept-treated group (P = 0.864).

On the other hand, we observe a different pattern in the evaluation of CMT and CMV during the study (Table 6). At baseline, in the ranibizumab-treated group CMT was 483.45 (+/-142.13) μm and in the aflibercept-treated group CMT was 419.46 (+/-104.61) μm (P<0.001). This difference can be explained by the different number of naïve patients in the ranibizumab and aflibercept groups. Even if differences still remain at 4^th^ month visit, at the end of the follow up they disappear, since the final CMT of the ranibizumab-treated group was 377.06 (+/-116.59) μm, and that of aflibercept-treated group was 365.56 (+/-105.22) μm (P = 0.459). When we compare naïve and non-naïve patients we can appreciate that in the naïve group the change of CMT is much greater than in the other group. At the beginning of the study the naïve patients CMT was 480.73 (+/-142.72) μm and the CMT of non-naïve patients was 430.31 (+/-112.67) μm (P<0.001) and at the end of the study this difference disappeared, being CMT in naïve patients 362.56 (+/-96.01) μm and 382.19 (+/-125.88) μm (P = 0.800) in non-naïve patients. This is explained by the greater amount of naïve patients in the ranibizumab-treated group and the greater amount of non-naïve patients in the aflibercept-treated group.

When performing the multivariate regression, the differences disappeared as can be seen in Table 4. The factors that affected final BCVA the most were previous treatment with bevacizumab, macular laser, female gender and, especially, baseline BCVA, which was determined as the most important one, and is the one adjusted in Table 4. Patients treated with bevacizumab and macular laser had worse BCVA at the end of our study, and this can be explained by the fact that these patients had a chronic DME and had received treatments for this outcome before the beginning of our study, so they were considered non-naïve, a fact that is linked to worse improvements in visual acuity [6,9,10,14,18]. The fact that female patients had a poorer BCVA prognosis in our study we put down to chance. No other study has documented this issue.

With regard to baseline BCVA as the most important factor determining final BCVA, in Protocol T, Wells et al [13], came to the same conclusion as we subsequently do in this study. When the initial loss of visual acuity is mild, the improvement is not as spectacular as when baseline BCVA is worse, even though patients with good visual acuity at the beginning end up with good BCVA, and patients with worse baseline BCVA end up with a final visual acuity that is not so good. They conclude that final BCVA is conditioned by baseline one [13]. In the first year of the study they found that aflibercept tended to be more effective than ranibizumab and bevacizumab in patients with a low baseline BCVA. However, we do not seem to find this difference in our own work. In one study performed in Egypt by Fouda et al, patients with low BCVA at baseline were evaluated [4]. They found no differences in terms of efficacy between ranibizumab and aflibercept, but they concluded that aflibercept-treated patients may need fewer injections. In our study, patients with good BCVA at baseline maintain their visual acuity, from 0.24 (+/- 0.78) logMAR to 0.22 (+/- 0.19) and those with bad BCVA at baseline improved, from 0.69 (+/- 0.32) logMAR to 0.51 (+/- 0.35) logMAR, but they did not attain the visual acuities of the other group (P<0.001). This issue has already been demonstrated in the RESTORE and RELIGHT trials as well [6,19].

Our patients received a mean of 5.77 (+/- 2.01) injections. The ranibizumab group received a mean of 5.56 (+/- 2.0) injections and the aflibercept group a mean of 6.07 (+/- 1.99) (P = 0.069). When we divided patients into naïve and non-naïve, we found that naïve patients received 5.83 (+/- 2.02) injections and non-naïve patients 5.71 (+/- 2.01) injections (P = 0.656). This is a lower number than patients in the randomized clinical trials such as the RESTORE study patients (6.8 injections) [6] or the Protocol I study (8–9 injections) [20], and also less than other studies performed in clinical practice, such as the one conducted by Patrao et al (7.2 injections) [1] or the one performed by Campos et al [2]. Other real world studies performed the same number of injections as our study, for instance the one performed by Fouda et al (5,62 injections in the aflibercept group and 6,02 in the ranibizumab group) [4].

Finally, we appreciate that the improvement in BCVA is less evident in our study than in other clinical trials [6,7,10,13], but it is comparable to other real world studies [1,4,14,15,21]. We attribute our results to the lower number of injections compared to other studies.

Moreover I would like to point the limitations of our study. Even real world data is interesting to our knowledge; this information is less likely generalizable. Protocols in real life studies are not as strict as we can see in randomized clinical trials or in prospective studies and there are several confounding factors that interfere with the results.

In conclusion, aflibercept and ranibizumab are effective drugs for DME treatment and, as previous data indicates, patients`baseline BCVA is the most important value for final BCVA. Moreover, having not been previously treated is a factor indicating a good prognosis, because patients who received another treatment before the study did not respond totally to it and can be classified as poor responders or chronic DME and they are more likely to not respond to anti-VEGF as well as naïve patients.
